# The Gender Gap in EHR Workload: A Comparative Analysis of Primary Care Physician In Basket Usage

**DOI:** 10.1007/s11606-025-09629-w

**Published:** 2025-05-29

**Authors:** Greta L. Branford, Matthew D. Bucala, Amy Hepper, Nicole M. Hadeed, Rebecca M. Northway, Michael J. Brenner

**Affiliations:** 1 Department of Internal Medicine, Division of General Medicine, University of Michigan Medical School, Ann Arbor, MI USA; 2 Department of Otolaryngology-Head and Neck Surgery, University of Michigan Medical School, Ann Arbor, MI USA; 3 Division of General Medicine, University of Michigan Medical Center, Ann Arbor, MI USA

**Keywords:** Patient portal messaging, Physician burnout, Gender disparities, Electronic health record, Workload, Emotional exhaustion, Gender equity

## Abstract

**Background:**

Gender disparities in emotional exhaustion and burnout are well-recognized, but few studies have assessed gender differences in EHR patient portal workload and implications for clinician well-being.

**Objective:**

To investigate gender differences in EHR in basket activity, workload, and burnout among physicians.

**Design:**

Observational analysis of EHR usage data and survey responses.

**Participants:**

406 physicians (43.1% men, 56.9% women) at a large academic medical center.

**Main Measures:**

Time spent on EHR activities, including in basket tasks, orders, and notes. Survey responses were collected on factors contributing to burnout, experiences with patient portal messages, and perceptions of EHR usability.

**Key Results:**

Women physicians spent significantly more time on orders (34.00 vs 28.39 min/day, *p* = 0.004) and notes (70.38 vs 53.17 min/day, *p* = 0.004) than men physicians. Women physicians were more likely to report that patient portal messages required more clinical assessment (OR = 1.60, 95% CI: 1.05–2.45, *p* = 0.0297) and contributed to burnout (OR = 1.76, 95% CI: 1.12–2.79, *p* = 0.0152). They were also more likely to receive negative messages from patients (OR = 1.61, 95% CI: 1.08–2.42, *p* = 0.0204). Interestingly, women physicians found EHR systems easier to learn (OR = 2.03, 95% CI: 1.37–3.02, *p* < .0001) and were less likely to feel EHRs inhibited quality care (OR = 0.57, 95% CI: 0.43–0.74, *p* < .0001) compared to men physicians.

**Conclusion:**

Gender disparities exist in EHR usage and patient communication, and these disparities are associated with increased burnout risk among women physicians. Effective leadership engagement is essential for optimizing EHR workflows, promoting equitable work practices, and implementing flexible work provisions that ensure safe and sustainable care delivery.

**Graphical Abstract:**

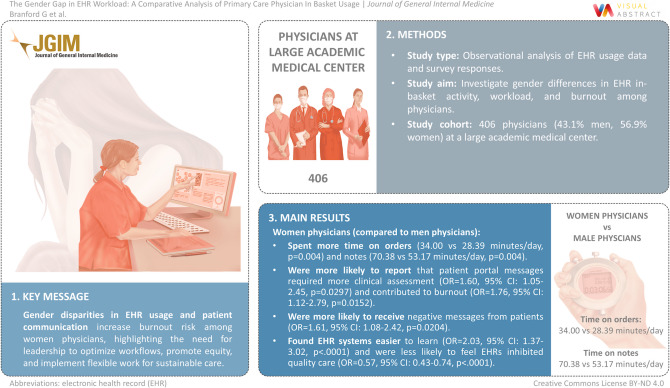

**Supplementary Information:**

The online version contains supplementary material available at 10.1007/s11606-025-09629-w.

## INTRODUCTION

The demands of electronic health record (EHR) systems have reshaped physicians'workloads, with the proliferation of inbox duties and patient-initiated messages significantly increasing administrative burdens.^1–4^ As expectations around timely communication intensify,^5,6^ physicians —already susceptible to longer work hours and burnout face an escalating risk of work-life imbalance.^7^ For women, limited schedule control,^8,9^ disproportionate domestic responsibilities,^10,11^ and substantial, often unrecognized service expectations compound these pressures.^11^ Additionally, gender bias, unequal compensation, and insufficient institutional support contribute to dissatisfaction and isolation for women physicians.^12,13^ These factors underscore the need to examine and address gender disparities in EHR workload to foster equitable and sustainable workforce practices in healthcare.

This study investigates gender disparities in EHR workload among healthcare professionals.^14,15^ We hypothesize that women physicians spend more time managing EHR tasks, such as reviewing and responding to patient messages, compared to their male counterparts.

EHR workload has been implicated as a major contributor to burnout, which has become increasingly prevalent in recent years,^16–18^ threatening healthcare professional well-being and patient care quality.^19,20^ Burnout is characterized by emotional exhaustion, depersonalization, and a diminished sense of personal accomplishment, leading to decreased job satisfaction, increased medical errors, and compromised patient safety.^21^ Numerous studies emphasize the need for systemic reforms to promote healthcare professionals’ well-being and mitigate these challenges.^22^ Research highlights the strong connection between physician well-being and retention. Emotional exhaustion, work-life imbalance, job dissatisfaction, and lack of institutional support are associated with academic physicians'intentions to leave their current positions.^23^ Women physicians, who constitute a substantial portion of the primary care workforce, are critical to maintaining healthcare delivery and are especially impacted by these burnout factors. The cost of physician burnout to the U.S. healthcare system is estimated at $4.6 billion annually due to physician turnover and reduced clinical hours.^24^ Addressing gender disparities in EHR workload is essential for reducing burnout, improving physician retention, and ensuring sustainable healthcare delivery systems.

Despite growing recognition of gender disparities in medicine and increasing EHR demands, little is known about how these factors intersect to affect physician workload and well-being. This study investigates gender differences in EHR usage patterns, hypothesizing that women physicians spend more time managing EHR tasks compared to their male counterparts. Understanding these disparities is critical for developing targeted interventions to promote equitable work practices and reduce physician burnout.

## METHODS

### Study Design and Participants

This study investigated gender differences in EHR in basket activity and physicians'perceptions of workload, efficiency, and gender dynamics. The study population consisted of 406 primary care/general medicine physicians (MD/DO) at a large academic medical center in the Midwest. Physicians were included if they had self-reported gender information (either women or men) available in their profiles or email signatures, regardless of FTE status. Residents and advanced practice providers were excluded from the analysis. This represents a subset of the total primary care physician population at our institution during the study period. Informed consent was obtained from all participants prior to their participation. The Institutional Review Board reviewed the study and determined that it was non-regulated and exempt from ongoing IRB oversight, as it did not meet the federal definition of human subjects research.

### Data Collection

Quantitative in basket activity data were collected via the EHR system's tracking tools, recording time spent and messages received by each physician over a one-year period (May 2023—April 2024). Additionally, the KLAS Arch Collaborative survey (full survey located in the Appendix) was distributed electronically to all primary care physicians at our institution via institutional email addresses. The survey gathered data on potential confounding factors like specialty, experience, practice setting, and EHR proficiency.^25^ All data were de-identified and securely stored. For both the EHR metrics and survey responses, incomplete data were excluded from the analysis, with less than 5% of the EHR dataset and less than 10% of the survey dataset excluded due to missing values. These exclusions ensured the quality of the analysis and did not significantly affect the representativeness of the study population.

### EHR Data Analysis

We employed a multivariable linear mixed-effects model to examine the relationship between gender, time, and other covariates on in basket time.^26,27^ This model incorporated both fixed effects (gender, time, and covariates like age, specialty, and years of experience) and random effects (individual subject variation). Gender and time were specified as fixed effects, with random intercepts and/or slopes for subjects. This approach allowed us to account for unbalanced data, missing observations, and individual variability while controlling for potential confounding factors. Message time was derived from Signal's in basket activity categorization to standardize workload comparisons. However,"time in in basket"only captures direct time spent in the in-basket interface, whereas in basket-related tasks (e.g., reviewing labs, placing orders, and documenting notes) are categorized separately, underestimating total workload (see Supplemental Table A1 for metric definitions).

To optimize cross-center comparisons, we normalized EHR usage data by full-time equivalent (FTE) status. FTE was chosen as the normalization metric because it reflects clinical effort and time commitment more directly than panel size, which may be influenced by factors such as case complexity, patient demographics, and team-based care models. Data on panel size or RVUs were not available for this study, which we acknowledge as a limitation. Normalization by FTE allowed for a consistent basis for comparing physicians with varying clinical workloads.

### Survey Data Analysis

Descriptive statistics summarized responses. A stratified analysis with gender-based sample weights addressed women's over-representation.^28,29^ Ordinal logistic regression examined gender-response relationships on Likert scales.^30,31^ Odds ratios and 95% CIs were calculated, with proportional odds assessed via Brant test.^32,33^

## RESULTS

### Survey

#### Participant Demographics

A total of 406 physicians (MD/DO) participated in the survey. Of these, 54.2% treated adult patients, 27.8% treated both adult and pediatric patients, and 13.1% treated only pediatric patients (Table 1). Regarding EHR experience, 6.5% had been using an EHR for less than 1 year, 9.5% for 1–2 years, 13.5% for 3–5 years, and 70.3% for 5 years or more. The sample consisted of 43.1% men and 56.9% women physicians.

**Table 1:** Survey Participant Demographics (*N* = 406)

Survey Data	
Characteristic	*N* (%)
Overall	406 (100%)
Patient Types	
Adult	220 (54.2%)
Adult and pediatric	113 (27.8%)
Pediatric	53 (13.1%)
Years Using EHR	
Less than 1 year	18 (6.5%)
Men	8 (44.4%)
Women	10 (55.6%)
1–2 years	26 (9.5%)
Men	11 (42.3%)
Women	15 (57.7%)
3–5 years	37 (13.5%)
Men	16 (43.2%)
Women	21 (56.8%)
5 years or more	192 (70.3%)
Men	83 (43.2%)
Women	109 (56.8%)
Gender Identity	
Men	175 (43.1%)
Women	231 (56.9%)

Data represents survey participants only (*N* = 406). Percentages for gender distribution within Years Using EHR represent the proportion of men and women in each experience category. Overall percentages may not total 100% due to rounding

#### Factors Contributing to Burnout from Patient Portal Messages

Regression analyses revealed no significant differences between men and women physicians in ranking the factors contributing to burnout from patient portal messages (Appendix Table B1). Both genders similarly ranked the importance of factors such as receiving messages on the same topic, message length, navigating multiple complex systems, high-priority messages, responding within 72 h, and messages that could have been handled by other staff.

#### Physician Experiences with the Patient Portal

Women physicians were significantly more likely than men physicians to agree that patient portal messages required more clinical assessment (OR = 1.60, 95% CI: 1.05–2.45, *p* = 0.029) and enhanced patient education (OR = 2.08, 95% CI: 1.38–3.15, *p* < 0.0001; Table 2). Additionally, women physicians were more likely to report receiving negative or demeaning messages from patients (OR = 1.61, 95% CI: 1.08–2.42, *p* = 0.020) and to feel that managing patient portal messages contributed to their burnout (OR = 1.76, 95% CI: 1.12–2.79, *p* = 0.015).

**Table 2:** Physician Experiences with the Patient Portal (*N* = 406)

Outcome	Gender	OR	95% CI	*P*-values
Considering your experience with the patient portal, please rate the following statements				
Messages about system issues	Men	-	-	
	Women	1.44	(0.92, 2.25)	0.106
Benefit from educating patients	Men	-	-	
	Women	1.46	(0.95, 2.26)	0.084
More clinical assessment	Men	-	-	
	Women	1.60	(1.05, 2.45)	0.029
Enhances patient education	Men	-	-	
	Women	2.08	(1.38, 3.15)	<.0001
Negative demeaning message	Men	-	-	
	Women	1.61	(1.08, 2.42)	0.020
Patient sends same requests	Men		-	
	Women	1.39	(0.92, 2.10)	0.121
Fosters better patient trust	Men	-	-	
	Women	0.75	(0.49, 1.13)	0.166
Prevents negative outcomes	Men	-	-	
	Women	0.71	(0.47, 1.07)	0.099
Enhanced patient engagement	Men	-	-	
	Women	1.44	(0.92, 2.25)	0.106
Contribute to burnout	Men	-	-	
	Women	1.76	(1.12, 2.79)	0.015

*OR* Odds Ratio; *CI* Confidence Interval. The ordinal logistic regression models estimate the odds ratios for women physicians compared to men physicians (reference category) for rating each statement higher on a Likert scale. An odds ratio greater than 1 indicates that women physicians are more likely to agree with the statement, while an odds ratio less than 1 indicates that women physicians are less likely to agree with the statement compared to men physicians

Importantly, time spent in notes, clinical review, and orders includes work initiated from in-basket messages, while in basket time only captures activities within the in basket interface itself. When providers click into encounters from messages to evaluate issues, look up information, place orders, or document responses, this time is recorded under the respective categories rather than in basket time

#### Burnout and Work-Related Stress

Managing patient portal messages did not significantly differ by gender in contributing to stress, burnout, or work-home conflict (Appendix Table C1).

#### Primary Contributors to Feelings of Burnout

Women physicians were significantly less likely than men physicians to cite a lack of personal control over their workload as a primary contributor to feelings of burnout (OR = 0.81, 95% CI: 0.67–0.99, *p* = 0.038; Table 3). In contrast, women physicians were more likely to identify a lack of effective teamwork as a primary contributor to burnout (OR = 1.24, 95% CI: 1.01–1.53, *p* = 0.044). Notably, women physicians were significantly less likely to feel that the EHR inhibited quality care compared to their men counterparts (OR = 0.57, 95% CI: 0.43–0.74, *p* < 0.0001).

**Table 3:** Primary Contributors to Feelings of Burnout Among Physicians by Gender (*N* = 406)

Outcome	Gender	OR	95% CI	*P*-value
What are the primary contributors to your feelings of burnout (if any)?				
No personal control over my workload	Men	-	-	-
	Women	0.81	(0.67, 0.99)	0.038
Lack of autonomy in my job	Men	-	-	-
	Women	0.95	(0.74, 1.21)	0.674
Chaotic work environment	Men	-	-	-
	Women	1.17	(0.96, 1.43)	0.119
Lack of effective teamwork	Men	-	-	-
	Women	1.24	(1.01, 1.53)	0.044
Lack of shared values	Men	-	-	-
	Women	0.93	(0.76, 1.14)	0.459
Too much bureaucratic tasks	Men	-	-	-
	Women	0.92	(0.76, 1.10)	0.349
Staffing shortages	Men	-	-	-
	Women	1.18	(0.99, 1.40)	0.067
After hours workload	Men	-	-	-
	Women	0.92	(0.74, 1.14)	0.448
EHR inhibits quality care	Men	-	-	-
	Women	0.57	(0.43, 0.74)	<.0001
EHR hurts efficiency	Men	-	-	-
	Women	0.88	(0.71, 1.10)	0.265
Lack of EHR training	Men	-	-	-
	Women	0.93	(0.69, 1.28)	0.670
Aggressive patients	Men	-	-	-
	Women	1.22	(0.99, 1.52)	0.068
COVID concerns	Men	-	-	-
	Women	1.14	(0.63, 2.18)	0.682
*Other	Men	-	-	-
	Women	1.27	(0.96, 1.70)	0.100

*OR* Odds Ratio; *CI* Confidence Interval. The ordinal logistic regression models estimate the odds ratios for women physicians compared to men physicians (reference category) for rating each statement higher on a Likert scale. An odds ratio greater than 1 indicates that women physicians are more likely to agree with the statement, while an odds ratio less than 1 indicates that women physicians are less likely to agree with the statement compared to men physicians

#### Physician Agreement with Statements about their EHR System

Women physicians were significantly more likely than men physicians to agree that their EHR system was easy to learn (OR = 2.03, 95% CI: 1.37–3.02, *p* < 0.0001; Table 4). No significant differences were observed between genders for other statements regarding EHR functionality, integration, response time, patient safety, or the ability to deliver high-quality, patient-centered care.

**Table 4:** Physician Agreement with Statements About their EHR System by Gender (*N* = 406)

Outcome	Predictor	OR	95% CI	*P*-value
Do you agree with the following statements? This EHR…				
Enables me to deliver high-quality care	Men	-	-	
	Women	1.41	(0.94, 2.10)	0.091
Makes me as efficient as possible	Men	-	-	
	Women	0.74	(0.50, 1.10)	0.128
Is available when I need it	Men	-	-	
	Women	1.01	(0.67, 1.53)	0.953
Has the functionality for my specific specialty/clinical care focus	Men	-	-	
	Women	1.11	(0.75, 1.65)	0.596
Provides expected integration within our organization	Men	-	-	
	Women	1.20	(0.80, 1.80)	0.379
Provides expected integration with outside organizations	Men	-	-	
	Women	1.27	(0.86, 1.89)	0.234
Has the fast system response time I expect	Men	-	-	
	Women	1.40	(0.93, 2.09)	0.104
Is easy to learn	Men	-	-	
	Women	2.03	(1.37, 3.02)	<.0001
Has alerts that prevent care-delivery mistakes	Men	-	-	
	Women	1.47	(0.99, 2.20)	0.056
Keeps my patients safe	Men	-	-	
	Women	1.19	(0.70, 1.53)	0.383
Allows me to deliver patient-centered care	Men	-	-	
	Women	1.04	(0.69, 1.78)	0.855

*OR* Odds Ratio; *CI* Confidence Interval. The ordinal logistic regression models estimate the odds ratios for women physicians compared to men physicians (reference category) for rating each statement higher on a Likert scale. An odds ratio greater than 1 indicates that women physicians are more likely to agree with the statement, while an odds ratio less than 1 indicates that women physicians are less likely to agree with the statement compared to men physicians

### EHR Metrics

We analyzed gender differences in EHR activities over one year (May 2023—April 2024; Tables 5 and 6). Women physicians spent significantly more time on EHR tasks, accounting for 65–71% of total time across most metrics: inbasket activities (65.4%, 130,198 vs. 66,309 min), messages (65.0%, 48,399 vs. 25,999 min), orders (69.4%, 102,510 vs. 45,227 min), notes (71.4%, 211,392 vs. 84,709 min), and clinical review (67.8%, 117,393 vs. 55,644 min). Turnaround times remained similar between genders (women: 720 min, men: 999 min).

**Table 5:** Overall Time Spent on EHR Metrics by Gender (May 2023—April 2024)

EHR Data		
Metric	Women *N* (%)	Men *N* (%)
Time in in basket per day		
Count (%)	3,817 (65.4%)	2,021 (34.6%)
Total time (minutes)	130,198	66,309
In basket messages received per day		
Count (%)	949 (65.0%)	511 (35.0%)
Total time (minutes)	48,399	25,999
Turnaround time		
Count (%)	948 (65.0%)	510 (35.0%)
Total time (minutes)	720	999
Time in orders per day		
Count (%)	3,015 (65.4%)	1,593 (34.6%)
Total time (minutes)	102,510	45,227
Time in notes per day		
Count (%)	3,015 (65.4%)	1,593 (34.6%)
Total time (minutes)	211,392	84,709
Time in clinical review per day		
Count (%)	3,155 (65.4%)	1,670 (34.6%)
Total time (minutes)	117,393	55,644

^1^Time spent in in basket per day: Time spent by physicians managing their inbox and messages within the EHR system

^2^Turnaround time: The total time taken to complete a process or task from start to finish

^3^Time in orders per day: The amount of time spent on processing or handling orders received each day

^4^Time in notes per day: The amount of time dedicated to writing or reviewing notes per day

^5^Time in clinical review per day: The amount of time allocated for reviewing clinical cases or patient records per day

**Table 6:** Comparison of Time Spent on Various EHR Activities between Women and Men Physicians

Metric	Women Physicians	Men Physicians		
	Mean (SD)	Mean (SD)	Mean Difference (95% CI)	*P*-value
Time in in basket per day^1^	34.11 (12.61)	32.81 (12.61)	−1.30 (−4.59, 1.99)	0.441
In basket messages received per day	51.02 (17.88)	49.90 (17.88)	−1.13 (−6.53, 4.27)	0.684
Turnaround time^2^	0.76 (62.38)	1.96 (62.38)	1.20 (−6.43, 8.83)	0.758
Time in orders per day^3^	34.00 (14.45)	28.39 (14.45)	−5.60 (−9.36, −1.85)	0.004
Time in notes per day^4^	70.38 (40.50)	53.17 (40.50)	−17.21 (−28.76, −5.66)	0.004
Time in clinical review per day^5^	37.26 (15.81)	33.20 (15.81)	−4.06 (−8.34, 0.22)	0.065
Time outside scheduled hours^6^	48.41 (32.14)	44.40 (32.14)	−4.01 (−18.08, 10.06)	0.555

^1^Time spent in in basket per day: Time spent by physicians managing their inbox and messages within the EHR system

^2^Turnaround time: Mean daily time taken to complete a process or task from start to finish (in minutes/day)

^3^Time in orders per day: The amount of time spent on processing or handling orders received each day

^4^Time in notes per day: The amount of time dedicated to writing or reviewing notes per day

^5^Time in clinical review per day: The amount of time allocated for reviewing clinical cases or patient records per day

Mixed-effects model analysis showed women physicians spent significantly more time on orders (34.0 vs. 28.3 min/day; difference: 5.6 min, 95% CI: −9.36, −1.85) and notes (70.3 vs. 53.1 min/day; difference: 17.2 min, 95% CI: −28.7, −5.6) compared to men (both *p* = 0.004; Table 2). Time categorization in the EHR system attributes work initiated from in basket messages to notes, orders, and clinical review rather than in basket time.

Despite no significant gender differences in"time in in basket"(*p* = 0.441), messages received (*p* = 0.684), or turnaround time (*p* = 0.758), women physicians spent more time on in basket-related tasks (orders, notes), which are categorized separately in the EHR system.in basket messages received (51.0 vs. 49.9 min/day, *p* = 0.684), or turnaround time (0.76 vs. 1.96 min, *p* = 0.758). Time spent on clinical review approached significance (37.2 vs. 33.2 min/day, *p* = 0.065), with women physicians spending slightly more time on this activity.

### Data Triangulation

In contrast to the aggregate metrics in Table 5, Table 6 focuses on individual-level differences in mean daily time spent on EHR activities, adjusted for covariates using a linear mixed-effects model. This analysis revealed no significant differences in average daily time spent on in basket activities or messages by gender, but significant differences were observed in time spent on orders (*p* = 0.004) and notes (*p* = 0.004) These findings align with the survey results, which showed that women physicians were significantly more likely than men physicians to agree that patient portal messages required more clinical assessment (OR = 1.60, 95% CI: 1.05–2.45, *p* = 0.029) and feel that managing patient portal messages contributed to their burnout (OR = 1.76, 95% CI: 1.12–2.79, *p* = 0.015). (Table 3). The convergence of these findings suggests that women physicians may spend more time on orders and notes due to the increased clinical assessment and patient education required for managing patient portal messages.

## DISCUSSION

Our study documents gender disparities in EHR workload and patient portal management, confirming and extending previous findings.^34,35^ These differences highlight the need for systemic changes to address increased workload and burnout risk among women physicians.

### Gender Differences in EHR Usage, Workload, and Burnout

Our analysis of EHR metrics revealed that women physicians consistently spent more time on various EHR activities compared to their men counterparts. Notably, women physicians spent significantly more time on orders (*p* = 0.004) and notes (*p* = 0.004) per day. However, time in in basket itself did not differ by gender (*p* = 0.441), underscoring how EHR classification may obscure the burden of in basket work. This finding aligns with previous studies suggesting that women physicians tend to spend more time on EHR tasks and patient communication.^36,37^ The additional time spent on these activities may contribute to longer work hours and an increased risk of burnout among women physicians. ^38,39^ Age and career stage may also be a factor, as younger physicians often report higher burnout rates than their older counterparts.^40,41^ These findings suggest a role for exploring the potential intersection between gender with career stage, with a focus on early-career women physicians.

The gender disparity in EHR usage may stem from multiple factors, including societal expectations, communication styles, and patient demographics.^42,43^ Women physicians often spend more time on patient communication and documentation, possibly due to differing practice styles or patient expectations.^44–46^ Because the EHR system attributes tasks like order entry and documentation to separate categories, much of the work initiated from in basket messages is not counted as in basket time, further obscuring true workload differences. Despite spending more time on EHR tasks, women physicians maintained equal turnaround times despite longer EHR task durations, suggesting efficient management of a higher workload. These differences likely result from complex societal, professional, and organizational factors affecting genders differently,^47^ rather than inherent gender traits. Further research is needed to disentangle these factors and develop equitable interventions for workload distribution and physician support.

Several factors contribute to the gender gap in EHR workload: women patients send more portal messages and preferentially choose women physicians,^48^ patients with multiple diagnoses generate more messages,^49^ and COVID-19 increased messaging burden disproportionately for women physicians.^50^ Women physicians'reports of requiring more clinical assessment and documentation time align with previous studies showing increased EHR task burden.^51–54^

Women physicians were more likely to report that patient portal messages contributed to their burnout, consistent with prior research on the deleterious effects of excessive EHR burden.^47,55,56^ Our usage data showed that women physicians spend greater time spent on these activities, emphasizing the complex relationship between workload, gender, and burnout in healthcare settings. Previous research has shown that women physicians face a higher risk of burnout compared to their men counterparts,^57,58^ and our findings of additional time spent on EHR tasks and patient communication provide one possible mechanism for this propensity.^59^ While our study focused on quantitative differences in EHR usage by gender, relevant qualitative research has examined the broader implications of EHR use on physician workload. For example, Attipoe et al. explored the pressures and opportunities associated with EHR work outside of regular hours, highlighting the dual impact of flexibility in enabling work-life integration while also contributing to potential burnout.^60^ Future research could build on these insights to explore how such pressures differ by gender and intersect with systemic workload disparities.

### Organizational Culture and Support

Our survey revealed that women physicians were significantly more likely to report receiving negative or demeaning messages from patients. This finding aligns with previous studies showing that women physicians are more likely to experience discrimination and harassment in the workplace.^61,62^ Such experiences can contribute to feelings of isolation and dissatisfaction, potentially exacerbating the risk of burnout.^63^ Interestingly, women physicians in our study were less likely to cite a lack of personal control over their workload as a primary contributor to burnout. However, they were more likely to identify a lack of effective teamwork as a primary contributor. These results suggest that organizational factors, particularly team dynamics, play a crucial role in the work experiences of women physicians. This aligns with previous research emphasizing the importance of organizational culture in addressing gender disparities and promoting physician well-being.^64,65^

Addressing these disparities requires a multifaceted approach. Healthcare organizations should consider reviewing panel sizes and distribution to ensure fair workloads. In addition, lowering productivity requirements and transitioning to value-based care models may alleviate administrative burdens and promote equitable work practices. Value-based care, which prioritizes patient outcomes over service volume, could help reduce time-intensive EHR documentation and foster more balanced workloads across providers. Organizational-level interventions such as flexible work arrangements, mentorship programs, and diversity and inclusion initiatives can create a more supportive work environment for all healthcare professionals, ensuring equitable work experience for women physicians.^66–68^ Furthermore, transparency in the allocation of resources and periodic reassessment of workload distribution are essential to maintaining parity and addressing emerging imbalances as healthcare demands evolve.

We found that women physicians were more likely to agree that their EHR system was easy to learn. Additionally, women physicians were less likely to feel that the EHR inhibited quality care compared to their men counterparts. These findings are intriguing, given that women physicians spent more time on EHR tasks. It's possible that women physicians'increased engagement with the EHR system leads to greater familiarity and perceived ease of use, despite the additional time investment.^69–72^ Institutions need to find ways to recognize and value this additional time and effort, as more thorough patient care and documentation could contribute to improved patient experience or care quality. Men physicians may have more often required a billable visit for complex patient inquiries or delegate administrative tasks to other staff. Given the impact of burnout-related turnover and reduced clinical hours,^73–76^ organization should prioritize optimizing EHR workflows, leveraging artificial intelligence, and ensuring equitable administrative support and staffing.^77,78^ While still evolving, these technologies hold promise for reducing documentation burdens and addressing workload disparities, but further research is needed to evaluate their impact.

### Limitations

This study, conducted at a single Midwest academic medical center with 406 physicians, has limitations in generalizability and specialty representation. Potential biases include the method of gender classification, overrepresentation of women physicians, and reliance on self-reported data. Additionally, we did not have access to data on patient types or years of EHR use, which may influence workload and efficiency metrics. Including these variables in future studies could provide a more comprehensive understanding of the factors contributing to gender differences in EHR usage. The cross-sectional design and one-year period preclude causal inferences and long-term trend analysis. Despite these constraints, the study offers significant insights into gender disparities in physician workload and EHR usage at a single institution. Its strengths include data triangulation, year-long data collection, and inclusion of various specialties. The findings address a critical aspect of healthcare and offer meaningful implications for organizations and policymakers, providing a foundation for future research and policy development in healthcare management.

### Implications and Future Directions

Understanding the gender gap in EHR workload can inform innovations to improve physician well-being and healthcare system efficiency. Future research should include longitudinal studies tracking these disparities over time and qualitative investigations of how practice styles and patient expectations influence EHR usage patterns.^79^ Additionally, examining the impact of gender differences on career trajectories and patient outcomes will help develop targeted interventions.^80,81^

## CONCLUSION

Our study demonstrates significant gender disparities in EHR workload at a large academic medical center, with women physicians spending more time on EHR activities despite reporting greater system proficiency. These findings highlight the need for targeted interventions to optimize workflows and promote equitable work distribution. Future research should examine the underlying causes and impacts of these gender differences to inform effective solutions.

## Supplementary Information

Below is the link to the electronic supplementary material.

Supplementary file1 (DOCX 3928 KB)

## Data Availability

The data supporting this study are not publicly available due to institutional policies and participant confidentiality.
