# Supplementary material for: The Gender Gap in EHR Workload: A Comparative Analysis of Primary Care Physician In Basket Usage
Source: J Gen Intern Med. 2025 May 29;40(10):2255–64. doi: 10.1007/s11606-025-09629-w (PMC12344033; doi:10.1007/s11606-025-09629-w)
Supplement: Supplementary file 1 — Supplementary file1 (DOCX 3928 KB) [file 11606_2025_9629_MOESM1_ESM.docx]

**APPENDIX**

**Table of Contents**

*Appendix methods*

Methods for Integrating Findings from In Basket Activity and Survey Data Analysis 35

*Supplemental tables*

Table A1. Definitions of EHR Metrics and Categorization of In-Basket Work 36

Table B1. Factors contributing to burnout from patient portal messages 37

Table C1. Burnout and work-related stress among physicians by gender 38

KLAS Survey Instrument: Michigan Medicine EHR Experience Survey 2023 39-66

**Appendix Methods**

*Data Triangulation*

Data triangulation was employed to gain a comprehensive understanding of gender differences in in basket activity and physicians' perceptions by comparing and integrating the findings from the in-basket activity data analysis and the survey data analysis.^[[1]](#footnote-1)^ The convergence or divergence between the two datasets was examined, looking for areas where the findings from the in basket activity analysis aligned with or contradicted the results from the survey data analysis. It's important to note that while the EHR data analysis included 174 selected primary care physicians, the survey responses were anonymous and may not represent the exact same group. This limitation was considered during our data triangulation process.

| **Table A1.** Definitions of EHR Metrics and Categorization of In-Basket Work   \| **Metric** \| **Definition** \| \| --- \| --- \| \| **Time in in-basket** \| Measures time spent within the in basket environment itself. However, it underestimates the total time spent on in basket-related tasks since activities like reviewing results, refilling Rx and answering complex messages within the in basket are categorized by the EHR vendor’s User Activity Log separately under clinical review, orders, or notes. This distinction explains why in basket time alone does not fully reflect the burden of patient messaging, and in fact misses the most complex activities in this space. \| \| **Time in EHR** \| A broader metric that includes all time spent within the EHR, such as in-basket use, data review, note-writing, order entry, and other clinical activities. It serves as a comprehensive measure of overall EHR engagement. \| \| **Total time required to complete in-basket work** \| Epic’s analytics do not directly measure this metric, so our study does not report quantitative data. Instead, qualitative insights in the manuscript highlight the perceived burden of in-basket tasks. The lack of direct comparison between qualitative and quantitative findings is acknowledged as a limitation in the revised manuscript. \|   **Note:** EHR system categorizations may underestimate the total burden of in-basket work by classifying related activities (e.g., reviewing results, placing orders, documenting notes) under separate categories. As a result, "time in in-basket" does not fully capture the workload associated with patient messaging and clinical decision-making.  **Table B1.** *Factors contributing to burnout from patient portal messages (N406)* |
| --- | --- | --- | --- | --- | --- | --- | --- | --- |

| **Outcome** | **Gender** | **OR** | **95% CI** | **P-value** |
| --- | --- | --- | --- | --- |
| Rank the following factors in order of their contribution to your feelings of burnout |  |  |  |  |
| Same topic messages | Male | - | - |  |
|  | Female | 0.83 | (0.64, 1.07) | 0.153 |
| Message length | Male | - | - |  |
|  | Female | 1.07 | (0.83, 1.36) | 0.608 |
| Multiple complex systems | Male | - | - |  |
|  | Female | 1.0 | (0.77, 1.30) | 0.985 |
| High priority messages | Male | - | - |  |
|  | Female | 1.07 | (0.83, 1.36) | 0.587 |
| Respond within 72 hours | Male | - | - |  |
|  | Female | 0.96 | (0.75, 1.23) | 0.767 |
| Could have been handled by other staff | Male | - | - |  |
|  | Female | 1.14 | (0.88, 1.46) | 0.323 |

*OR = Odds Ratio; CI = Confidence Interval. The ordinal logistic regression models estimate the odds ratios for female physicians compared to male physicians (reference category) for ranking each factor higher in terms of its contribution to burnout. An odds ratio greater than 1 indicates that female physicians are more likely to rank the factor higher, while an odds ratio less than 1 indicates that female physicians are less likely to rank the factor higher compared to male physicians.*

**Table C1.** *Burnout and work-related stress among physicians by gender (N406)*

| **Outcome** | **Gender** | **OR** | **95% CI** | **P-value** |
| --- | --- | --- | --- | --- |
| Does your time in the EHR after scheduled clinic hours create work-home conflict? | Male | - | - |  |
|  | Female | 0.97 | (0.68, 1.39) | 0.881 |
| To what extent does managing patient portal messages contribute to your work-related stress or burnout | Male | - | - |  |
|  | Female | 0.87 | (0.69, 1.11) | 0.275 |
| Level of burnout (5 ordinal categories, from 'no symptoms' to 'completely burned out') | Male | - | - |  |
|  | Female | 1.28 | (0.94, 1.73) | 0.115 |

*Note: OR = Odds Ratio; CI = Confidence Interval. The ordinal logistic regression models estimate the odds ratios for female physicians compared to male physicians (reference category) for rating each statement higher on a Likert scale. An odds ratio greater than 1 indicates that female physicians are more likely to agree with the statement, while an odds ratio less than 1 indicates that female physicians are less likely to agree with the statement compared to male physicians.*

**KLAS Survey Instrument**

**Michigan Medicine EHR Experience Survey 2023**

**Start of Block: Core Questions**

Q134 All responses are being collected by KLAS Research. While KLAS will need to identify you by name for internal purposes and for the integrity of the study, your identity will not be released to your sponsoring organization (see above) with the results of this survey without your permission. To learn more about how we process and protect your personal data, you may view our Privacy Policy.  

K102 **Contact information**
*Required

- First name* __________________________________________________
- Last name* __________________________________________________
- Email address __________________________________________________

| 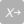 |
| --- |

K103 **Clinical background***

- Practicing physician (MD/DO)
- Physician resident or fellow
- Dentist (DDS/DMD)
- Clinical nurse specialist (CNS)
- CRNA
- Midwife
- Nurse practitioner
- Physician assistant
- Licensed practical nurse (LPN/LVN)
- Medical assistant (MA)
- Nursing assistant
- Registered nurse (RN)
- Unit clerk
- Behavioral therapist
- Dietician/Nutritionist
- Occupational therapist
- Physical therapist
- Pharmacist
- Psychologist
- Respiratory therapist
- Social worker
- Speech therapist
- Technician (Lab, Pharmacy, Radiology, etc.)
- Other (please specify) __________________________________________________

| Page Break |  |
| --- | --- |

K202 **Area of clinical focus (specialty)** (select one)

- Adolescent medicine
- Allergy and immunology
- Anesthesiology/pain medicine
- Cardiology
- Cardiothoracic surgery
- Complex care
- Critical care medicine
- Dentistry
- Dermatology
- Emergency medicine
- Endocrinology
- Family medicine
- Gastroenterology
- General surgery
- Genetics
- Geriatrics
- Gynecology and obstetrics
- Hematology/oncology
- Hospice/palliative medicine
- Hospital medicine
- Infectious disease
- Informatics
- Internal medicine
- Internal medicine/Pediatrics
- Interventional radiology
- Neonatology
- Nephrology
- Neurology
- Neurosurgery
- Ophthalmology
- Oral and maxillofacial surgery
- Orthopedics
- Otorhinolaryngology
- Pathology (anatomic)
- Pathology (clinical)
- Pediatrics
- Physical medicine and rehabilitation
- Plastic, reconstructive, and aesthetic surgery
- Podiatric medicine
- Psychiatry
- Psychology
- Pulmonology
- Radiology
- Rheumatology
- Sports medicine
- Urology
- Other

K203 Other (please specify)

________________________________________________________________

K204 **Area of nursing focus** (select one)

- Adult acute care
- Adult critical care
- Ambulatory
- Behavioral health
- Case/care management
- CSR
- Dialysis
- Education
- Emergency care/survival flight
- Home care
- Infusion center
- Labor and delivery
- Leadership
- NICU
- Oncology
- Pain management
- Pediatric acute care
- Perinatal (mother/baby)
- Perioperative
- Peds critical care (PICU, PTCU)
- Procedural
- Radiology/IR
- Research
- Surgical (OR)
- Transplant
- Vascular access
- Wound ostomy
- Other

K205 Other (please specify)

________________________________________________________________

K104 ***In which setting do you primarily work?**

- Ambulatory care
- Inpatient care
- Inpatient and ambulatory care
- Emergency department
- Other (please specify) __________________________________________________

| Page Break |  |
| --- | --- |

K301 **Do you agree with the following statements?**
**This EHR...**

|  | Strongly disagree | Disagree | Neither agree nor disagree | Agree | Strongly agree | Not applicable |
| --- | --- | --- | --- | --- | --- | --- |
| ...enables me to deliver **high-quality care** |  |  |  |  |  |  |
| ...makes me as **efficient as possible** |  |  |  |  |  |  |
| ...is **available when I need it** (has almost no downtime) |  |  |  |  |  |  |
| ...has the **functionality** for my specific specialty/clinical care focus |  |  |  |  |  |  |
| ...provides expected **integration within our organization** |  |  |  |  |  |  |
| ...provides expected **integration with outside organizations** |  |  |  |  |  |  |
| ...has the fast **system response time** I expect |  |  |  |  |  |  |
| ...is **easy to learn** |  |  |  |  |  |  |
| ...has **alerts that prevent care-delivery mistakes** |  |  |  |  |  |  |
| ...keeps my **patients safe** |  |  |  |  |  |  |
| ...allows me to deliver **patient-centered care** |  |  |  |  |  |  |

K309 **Do you agree?**

|  | Strongly disagree | Disagree | Neither agree nor disagree | Agree | Strongly agree |
| --- | --- | --- | --- | --- | --- |
| My clinical practice is enhanced through the use of this EHR |  |  |  |  |  |

K207 **Do you agree?**

|  | Strongly disagree | Disagree | Neither agree nor disagree | Agree | Strongly agree | Have not participated | Did not know this was offered |
| --- | --- | --- | --- | --- | --- | --- | --- |
| My **initial training** prepared me well to use this EHR |  |  |  |  |  |  |  |
| Overall, **ongoing EHR training/education** is helpful and effective |  |  |  |  |  |  |  |
| I was trained on how to use the EHR for **workflows specific to my specialty** and/or practice area |  |  |  |  |  |  |  |

K306 **Do you agree with these statements?**

|  | Strongly disagree | Disagree | Neither agree nor disagree | Agree | Strongly agree |
| --- | --- | --- | --- | --- | --- |
| Our **EHR vendor** has designed a high-quality EHR |  |  |  |  |  |
| Our **organization** has done a great job of implementing, training on, and supporting the EHR |  |  |  |  |  |
| I have **personally** done a great job of learning the EHR system so that I can be successful |  |  |  |  |  |

| Page Break |  |
| --- | --- |

K401 **Using your own definition of burnout, select one of the answers below:**

- I enjoy my work and have no symptoms of burnout
- I am under stress and don’t always have as much energy as I used to, but I don’t feel burned out
- I am definitely burning out and have one or more symptoms of burnout (e.g., emotional exhaustion)
- The symptoms of burnout that I am experiencing won’t go away, and I think about work frustrations a lot
- I feel completely burned out, and I am at the point where I may need to seek help

If you are experiencing symptoms of distress and are considering harming yourself, please contact the national suicide lifeline by calling 988 or contact your Call Office of Counseling and Workplace Resilience 734-763-5409 or email counseling@med.umich.edu. 
 
Or visit the Burnout Toolkit website here.

| Page Break |  |
| --- | --- |

K402 **What are the primary contributors to your feelings of burnout (if any)?**
(Select all that apply)

- No personal control over my workload (working too many hours)
- Lack of autonomy in my job
- Chaotic work environment
- Lack of effective teamwork in my organization
- Lack of shared values with organization leadership
- Too much time spent on bureaucratic tasks
- Staffing shortages
- After-hours workload
- EHR or other IT tools inhibit my ability to deliver quality care
- EHR or other IT tools hurt my efficiency
- Lack of training/proficiency on EHR or other IT tools
- Aggressive and/or demeaning patients
- Work-related concerns due to COVID-19 (please specify) __________________________________________________
- Other (please specify) __________________________________________________

K403 **Within the next two years, how likely are you to leave your organization?**
(Your answer to this question **will remain anonymous** even if you agree to share your name with your organization.)

- Very unlikely
- Unlikely
- Neither unlikely nor likely
- Likely
- Very likely

UI71019215 **You have selected that you are either likely or very likely to leave your organization within the next two years. Please explain why.**

________________________________________________________________

________________________________________________________________

________________________________________________________________

________________________________________________________________

________________________________________________________________

**End of Block: Core Questions**

**Start of Block: EHR Education**

**EHR Education**

K208 **Would you like more EHR education/training?**

- Yes
- No

K207 **Do you agree?**

|  | Strongly disagree | Disagree | Neither agree nor disagree | Agree | Strongly agree | Did not know this was offered | Not applicable |
| --- | --- | --- | --- | --- | --- | --- | --- |
| **Tip sheets and self-directed online training** are helpful and effective |  |  |  |  |  |  |  |
| **In-person training** is helpful and effective |  |  |  |  |  |  |  |
| **Virtual instructor-led EHR training/education** has sufficiently met my needs |  |  |  |  |  |  |  |

K225 **Please rate your participation in and the usefulness of the following training and/or MiChart information/communication**

|  | Have used and found useful | Have used, but was not useful | Have not used, but I want to | Have not used and have no desire to use | Not applicable |
| --- | --- | --- | --- | --- | --- |
| **Scheduled** one-on-one training |  |  |  |  |  |
| **Impromptu** one-on-one training (e.g., rounding visit) |  |  |  |  |  |
| Training in a department meeting |  |  |  |  |  |
| **Self-directed** virtual training (e.g., Cornerstone modules, e-learnings) |  |  |  |  |  |
| Epic certified Power User classes |  |  |  |  |  |
| Boost Program training session |  |  |  |  |  |
| Home for Dinner 2 day CME course |  |  |  |  |  |
| Handout posted on unit (e.g., MiChart Minute) |  |  |  |  |  |
| TellAll or other email updates |  |  |  |  |  |
| Direct email or in-person communcation with provider champion or ACMIO |  |  |  |  |  |
| Direct contact with Nursing Informatics |  |  |  |  |  |
| Other (please specify) |  |  |  |  |  |

K228 **You've indicated that training was useful to you. How specifically did you benefit?**

________________________________________________________________

________________________________________________________________

________________________________________________________________

________________________________________________________________

________________________________________________________________

| Page Break |  |
| --- | --- |

**End of Block: EHR Education**

**Start of Block: EHR Improving Care**

Q144 **EHR Improving Care**

K311 **Please rank the areas of the EHR that need the most improvement/optimization?**
Rank in order where 1 is the biggest problem

| Need improvement |
| --- |
| ______ Clinical information review |
| ______ Care plans |
| ______ Discharge |
| ______ Flowsheets |
| ______ MAR |
| ______ Navigator/menu |
| ______ Notes |
| ______ Orders |
| ______ Patient education |
| ______ Brain/worklists |
| ______ Logging in to the EHR |
| ______ Secure chat |
| ______ Rover |

UI295231 **Please rank the areas of the EHR that you have seen the most improvement/optimization on in the last 12 months?**
Rank in order where 1 is the biggest problem

| Most improvement |
| --- |
| ______ Clinical information review |
| ______ Care plans |
| ______ Discharge |
| ______ Flowsheets |
| ______ MAR |
| ______ Navigator/menu |
| ______ Notes |
| ______ Orders |
| ______ Patient education |
| ______ Worklists/care compass |
| ______ Logging in to the EHR |
| ______ Brain |
| ______ Secure chat |
| ______ Rover |

K320 **Which of the following are examples of your issues with the EHR’s availability or slow response time? (Select all that apply)**

- Slow loading of elements in the EHR
- Unplanned downtime
- Issues with hardware (including laptops, computers, mouse, keyboard, carts, etc.)
- Slow login process
- Other (please specify) __________________________________________________

UI50921231 **Does your time in the EHR after scheduled clinic hours create work-home conflict?**

- Never
- Rarely
- Ocasionally
- Frequently
- Daily

U150921232 **Do you agree?**

|  | Strongly disagree | Disagree | Neither agree nor disagree | Agree | Strongly agree |
| --- | --- | --- | --- | --- | --- |
| HITS is responsive to customizations that help my area. |  |  |  |  |  |
| I have utilized MiChart Request (top R corner MiChart) to submit a concern or to request an enhancement. |  |  |  |  |  |
| I have a Super User, Power User, Provider Champion, ACMIO or trainer that I can reach out to with suggestions or questions. |  |  |  |  |  |
| Michigan Medicine leadership is effective in promoting ‘top of license’ work and empowering people of all levels to help with patient care as appropriate. |  |  |  |  |  |
| Michigan Medicine leadership is responsive to concerns of in basket burden. |  |  |  |  |  |
| Michigan Medicine staffs appropriately to handle patient volumes |  |  |  |  |  |

UI295232 **Do you use the MiChart In Basket?**

- Yes
- No

UI2818232 **Considering your experience with the patient portal, please rate the following statements:**

|  | Strongly disagree | Disagree | Neither agree nor disagree | Agree | Strongly agree |
| --- | --- | --- | --- | --- | --- |
| The portal has enhanced patient engagement, encouraging them to take a more proactive role in their healthcare. |  |  |  |  |  |
| The portal has been instrumental in preventing or avoiding negative outcomes in patient care. |  |  |  |  |  |
| The portal has fostered greater trust between patients and providers. |  |  |  |  |  |
| I have experienced situations where patients send the same request to multiple providers via the portal, leading to confusion or duplicated efforts. |  |  |  |  |  |
| I have received messages through the portal which were negative, demeaning and caused me distress. |  |  |  |  |  |
| The portal effectively enhances patient education by allowing easier sharing of tailored resources such as articles, videos, and brochures. |  |  |  |  |  |
| I have received messages from patients demanding care that I deemed inappropriate or felt required more clinical assessment before providing. |  |  |  |  |  |
| The portal would benefit from educating patients about appropriate uses and communications through the platform. |  |  |  |  |  |
| I have received messages from patients expressing frustration over system-related issues (such as access, wait times, or securing appointments with specialists) that fall outside of my direct control. |  |  |  |  |  |
| The volume and content of portal messages are a significant contributor to EHR-related burnout. |  |  |  |  |  |

UI50915231 **To what extent do you feel that the volume and management of messages through the patient portal contributes to your work-related stress or burnout?**

- Not at all
- Slightly
- Moderately
- Significantly
- Extremely

UI2818233 In regard to the content of messages received through the patient portal, please rank the following factors in order of their contribution to your feelings of burnout
 (Drag and drop options, with 1 being the most contributing factor and 7 being the least contributing factor)

______ Message length

______ Expectation of new, multiple or complex symptoms to be addressed through portal

______ Multiple messages within the same day on the same topic

______ Portal messages marked as High priority

______ Expectation to respond before 72 business hours

______ Receipt of cyber incivil, uncivil, or aggressive (IUA) messages

______ Incomplete information obtained before forwarding/forwarding messages that could have been dealt with by other staff person

K307
**Do you agree with these statements?**
 
This EHR...

|  | Strongly disagree | Disagree | Neither agree nor disagree | Agree | Strongly agree | Not applicable |
| --- | --- | --- | --- | --- | --- | --- |
| ...reduces duplicate orders of diagnostic tests and procedures |  |  |  |  |  |  |
| ...helps me achieve my workplace's safety goals (e.g., reduced rates of septic shock, CLABSI, CAUTI, falls, etc.) |  |  |  |  |  |  |

UI50922231 **Please sort the following possible uses of AI from most useful to least useful:**

______ Sorting messages/Forwarding messages by content

______ Answering phone/portal messages

______ Summarizing large amounts of information (to review external information or creating brief summary of an internal visit)

______ Drafts of letters to insurance companies to get prior authorization for medications and other services

______ Drafting letters, work notes or medical disability letters

______ Patient instructions

______ Chatbots/telecare

______ Customized medicine

______ Identifying research candidates

______ Custom scheduling based upon patient need

**End of Block: EHR Improving Care**

**Start of Block: Wellness**

Q111 **Wellness**

K405 **What do you do for patients that that could be safely done by other staff below your licensure?**
(select all that apply)

- Answer billing questions
- Refill medications that should be refilled by protocol or could have been pended by staff
- Basic medical concerns
- Completing paperwork (FMLA, school/work notes)
- Re-refer for same specialty/procedure or extend end-date
- Respond to duplicate messages
- Assist with scheduling and "where to put them" questions
- Instruct basic clinical care
- Room patients
- Assign or complete questionnaires
- Do education that could be completed by other staff (colon prep, injections, wound care, inhaler use, etc.)
- Other (please specify) __________________________________________________

**End of Block: Wellness**

**Start of Block: Demographic Information**

UI283231 **What is your gender identity?**

- Woman or girl
- Man or boy
- Trans woman
- Trans man
- Other
- Choose not to disclose
- Genderqueer
- Two-Spirit
- Nonbinary

K206 **Number of years you have used this EHR at your current organization**

|  | Less than 1 year | 1–2 years | 3–5 years | 6–9 years | 10+ years |
| --- | --- | --- | --- | --- | --- |
| Years using EHR |  |  |  |  |  |

K106 **What kind of patients do you care for?**

- Adults
- Pediatric patients
- Adults and pediatric patients

**End of Block: Demographic Information**

**Start of Block: Comments**

K501 **You have reported high satisfaction. What do you believe that you do differently from some of your peers that enables you to be highly successful with the EHR?**

________________________________________________________________

________________________________________________________________

________________________________________________________________

________________________________________________________________

________________________________________________________________

K502 **Provide specific examples of three things in our EHR that you would improve?**

- Change 1 __________________________________________________
- Change 2 __________________________________________________
- Change 3 __________________________________________________

K503 **Other related comments and/or concerns**

________________________________________________________________

________________________________________________________________

________________________________________________________________

________________________________________________________________

________________________________________________________________

K101 **Please indicate below whether you would like your organization to see your identity in connection with your responses.**   This allows us to enrich the data set to combine with Epic data and employment data to make your responses more meaningful. Commentary will not be indentified.

- Share my identity in connection with my responses with my sponsoring organization
- Do not share my identity in connection with my responses with my sponsoring organization

UI295233 **Would you like to be entered to win one of four $100 Amazon gift cards? If so, please enter your information below. Your responses will not be tied to your entry.**
 (If you take the survey more than once, your most recent completed survey is used. One entry per person.)

- First name __________________________________________________
- Last name __________________________________________________
- Email __________________________________________________

**End of Block: Comments**

1. . Thurmond VA. The point of triangulation. J Nurs Scholarsh. 2001;33(3):253-258. doi:10.1111/j.1547-5069.2001.00253. [↑](#footnote-ref-1)
